# Detection of the United States *Neisseria meningitidis* urethritis clade in the United Kingdom, August and December 2019 – emergence of multiple antibiotic resistance calls for vigilance

**DOI:** 10.2807/1560-7917.ES.2020.25.15.2000375

**Published:** 2020-04-16

**Authors:** Avril Brooks, Jay Lucidarme, Helen Campbell, Laura Campbell, Helen Fifer, Steve Gray, Gwenda Hughes, Aiswarya Lekshmi, Gabriel Schembri, Michael Rayment, Shamez N Ladhani, Mary E Ramsay, Ray Borrow

**Affiliations:** 1 Manchester Medical Microbiology Partnership, Manchester Royal Infirmary, Manchester, United Kingdom; 2 Meningococcal Reference Unit, Manchester Royal Infirmary, Public Health England, Manchester, United Kingdom; 3 Immunisation and Countermeasures Division, Public Health England, London, United Kingdom; 4 Blood Safety, Hepatitis, Sexually Transmitted Infections & HIV Division, Public Health England, London, United Kingdom; 5 The Northern Integrated Contraception, Sexual Health & HIV Service, Manchester Royal Infirmary, Manchester, United Kingdom; 6 Chelsea and Westminster Hospital NHS Foundation Trust, London, United Kingdom; 7 Paediatric Infectious Diseases Research Group, St George's University of London, London, United Kingdom

**Keywords:** Meningococcal, Urethritis, MSM, Heterosexual, antibiotic resistance, Invasive meningococcal disease

## Abstract

Since 2015 in the United States (US), the US *Neisseria meningitidis* urethritis clade (US_NmUC) has caused a large multistate outbreak of urethritis among heterosexual males. Its ‘parent’ strain caused numerous outbreaks of invasive meningococcal disease among men who have sex with men in Europe and North America. We highlight the arrival and dissemination of US_NmUC in the United Kingdom and the emergence of multiple antibiotic resistance. Surveillance systems should be developed that include anogenital meningococci.

From 2015, the United States (US) has experienced a large multistate outbreak of meningococcal urethritis among predominantly heterosexual males due to a novel non-serogroupable strain - the US *Neisseria meningitidis* urethritis clade (US_NmUC) [1]. As a precaution, in November 2018, Public Health England extended meningococcal surveillance to include anogenital meningococci retrieved from adults attending four sexual health clinics in London and four in Manchester. Among 72 isolates collected from symptomatic or NAAT-positive sexual health clinic attendees during 2019, two were found to belong to the US_NmUC strain. Here we describe the United Kingdom (UK) cases and corresponding isolates in the context of the US experience and consider the implications going forward.

## Isolation and phylogenetic analysis

The UK US_NmUC isolates were collected in Manchester (August 2019) and London (December 2019), respectively. They were both non-serogroupable with genotype P1.5–1,10–8:F3–6:ST-11 (cc11). Draft genomes were submitted to the PubMLST *Neisseria* database (IDs 72327 and 72325, respectively). Both were from rectal swabs from HIV-negative white British men who have sex with men (MSM) in their thirties/forties who reported unprotected oral and anal sex with three to six partners in the 3 months before being diagnosed. Both were users of pre-exposure prophylaxis (PrEP). There were no co-infections with gonorrhoea, chlamydia, HIV or syphilis. In addition, hepatitis B and C were ruled out in Manchester and *Mycoplasma genitalium* was ruled out in London. The London case had no history of travel in the previous 3 months. No recent travel was documented for the Manchester case, however, this did not form part of the sexual history. The case from Manchester was asymptomatic and had been successfully treated for gonococcal infection of the rectum 3 months prior (ceftriaxone 1 g, as per national guidelines [2]). The case from London had a 5-day history of rectal discharge and pain. On examination, proctoscopy was normal and microscopy showed mucus only. Neither patient had symptoms of urethritis. There were no known links between the patients.

On a phylogenetic analysis including 209 US US_NmUC isolates, obtained between 2013 and 2016 [1], the UK isolates formed a monophyletic clade suggesting a single introduction into the UK and subsequent dissemination (Figure). Both isolates were sensitive to cefotaxime and resistant to penicillin (MIC 0.38 mg/L) with the same *penA* allele (allele 316; reduced susceptibility-associated mutations F504L, A510V, I515V, H541N, I566V) as the US isolates. The isolate from London was resistant to ciprofloxacin (MIC 0.38 mg/L) having acquired part of a gonococcal DNA gyrase (*gyrA*) gene. It had also acquired a frameshifted gonococcal maltose phosphorylase gene making it unable to utilise maltose and giving it a carbohydrate utilisation profile more typically associated with gonococci (glucose positive (+), maltose negative (-), sucrose negative (-), lactose negative (-)). Both UK US_NmUC isolates possessed the same gonococcal *aniA* and *norB* alleles as the majority (196/209) of the US isolates [1]. Separate searches of the PubMLST *Neisseria* database (accessed 30/03/20) for all non-serogroupable cc11 genomes > 1 million base pairs (Mbp) (n = 247) and serogroup not stated cc11 genomes > 1 Mbp (n = 515) yielded 215 US_NmUC genomes all of which were from the US (n = 213) or the UK (n = 2) (data not shown).

**Figure:**
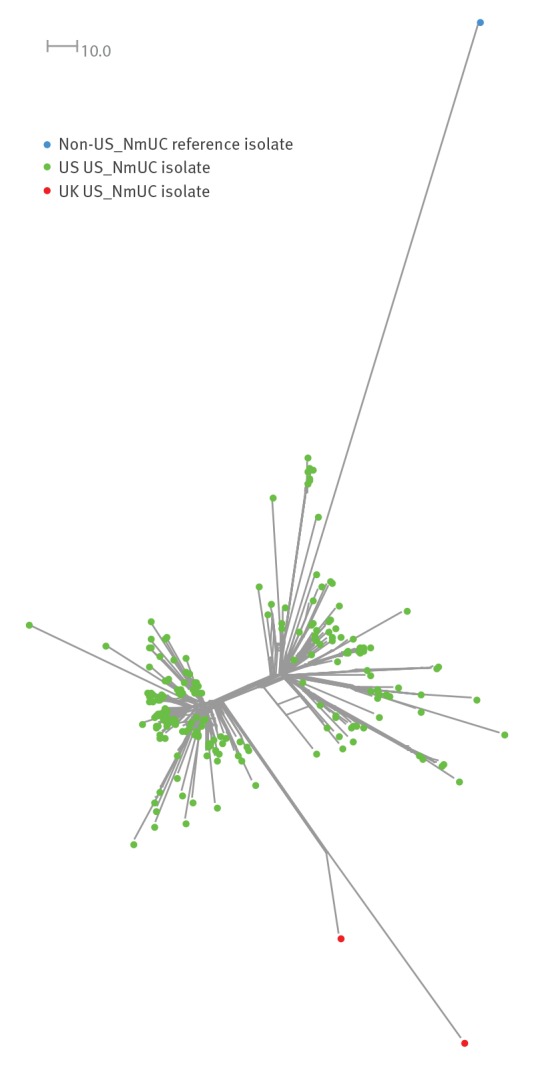
Neighbor-Net phylogenetic network analysis of United States (US) (n = 209) and United Kingdom (n = 2) US *Neisseria meningitidis* urethritis clade (US_NmUC) isolates

UK: United Kingdom; US: United States; US_NmUC: US *Neisseria meningitidis* urethritis clade.

The US US_NmUC isolates [1] formed two sub-strains. The UK isolates (PubMLST IDs 72325 and 72327) formed a monophyletic clade close to the evolutionarily more recent sub-strain as indicated by the relative position of the non-US_NmUC reference genome (M21273; PubMLST ID 47235). The reference genome (P1.5–1,10–8:F3–6: ST-2724 (cc11) is the complete circular genome of a closely related non-US_NmUC invasive serogroup C isolate and provided a reference for several aspects of the study by Retchless and colleagues [1] including the estimation of an evolutionary timeline for the US_NmUC strain [1]. In the present study, genomes were compared gene-by-gene in terms of 1,605 core genes using the PubMLST genome comparator tool [17]. The distance matrix generated was visualised using SplitsTree4 [18]. The scale bar represents the number of different loci among the 1,605 loci compared.

## Discussion and conclusions

*Neisseria meningitidis* is principally a harmless commensal of the oropharynx and is carried by ca 10% of the population at any time. Very rarely, meningococci cause invasive disease including meningitis and/or sepsis. Diverse meningococci have also been reported to infect the anogenital tract, albeit rarely, with or without clinically indistinguishable gonorrhoea-like symptoms such as urethritis, cervicitis or proctitis [3]. The association of the US_NmUC strain with urethritis represents a marked shift from this general diversity. In the US, in one clinic, it accounted for ca 20% of all Gram-negative, oxidase-positive diplococci on urethral culture from men [4]. It has also caused recurrent episodes of urethritis [5], neonatal conjunctivitis [6] and, despite being acapsulate, at least five cases of invasive disease, although the immune status of these patients was not reported [1]. The strain has been reported to be susceptible to ceftriaxone, azithromycin and ciprofloxacin and to have intermediate susceptibility to penicillin [4,6]. The US_NmUC strain exhibits several gonococcus-like adaptations to the anogential niche, including the ability to grow anaerobically having acquired gonococcal *aniA* and *norB* genes concerned with nitrite metabolism. The permanent lack of a capsule is due to the deletion of several capsule synthesis genes, including part of the serogroup C determinant gene [7].

The US_NmUC strain emerged around the year 2011 and forms part of a broader, predominantly serogroup C strain (PorA subtype P1.5–1,10–8: ST-11 complex) that, as well as causing invasive disease within the general community, has caused multiple outbreaks of serogroup C invasive disease among MSM in Europe and North America [8-10]. Phylogenetically, the isolates responsible for community cases and MSM outbreaks were broadly interspersed [11], however, discrete clustering of invasive MSM isolates and non-US_NmUC urethritis and proctitis isolates has been reported. Furthermore, all but one of the corresponding isolates had acquired an active *aniA* gene of meningococcal origin, in contrast to the gonococcal *aniA* of the US_NmUC strain [12]. Interestingly, the non-US_NmUC urethritis/proctitis isolates, like gonococci, did not express factor H-binding protein (fHbp) due to disruption of the *fhbp* gene. All but one of the invasive MSM isolates, meanwhile, retained their ability to express fHbp which is important for survival within the blood stream and, therefore, their ability to cause invasive disease [12]. Concerningly, the broader strain has a demonstrated propensity to escape both capsular and subcapsular meningococcal vaccines [13].

### Implications of the emergence of meningococci adapted to the anogenital niche

Aside from re-occurring invasive disease outbreaks among MSM, the association of this broad strain with sexual transmission, and its adaptation to the anogenital niche, pose several threats. Widespread acquisition of gonococcal DNA by US_NmUC strain isolates has already been demonstrated [1], raising the prospect of further acquisition of gonococcal antibiotic resistance determinants, as has now been observed with one of the UK isolates. Such genes may be passed onto more-typical meningococci, including hypervirulent lineages that typically reside within the oropharynx. Congenital complement deficiencies, excluding mannose-binding lectin deficiency, are estimated to affect approximately 0.03% of the general population [14] and increase the risk of invasive meningococcal disease by up to 10,000-fold. Similarly, the terminal complement pathway inhibitor, eculizumab, which is used to treat paroxysmal nocturnal haemoglobinuria and atypical haemolytic uraemic syndrome, is associated with up to 2,000-fold increased incidence of invasive meningococcal disease. This includes disease caused by unencapsulated strains, such as the US_NmUC strain, that would not normally cause disease in healthy individuals [15]. These individuals are recommended to receive meningococcal vaccination and long-term penicillin prophylaxis [16]. To account for the existence of relatively rare penicillin-resistant strains, ciprofloxacin may also be prescribed as a back-up should the patient experience symptoms of invasive meningococcal disease. The emergence of resistance to both penicillin and ciprofloxacin in one of the UK US_NmUC isolates, coupled with a propensity to escape licenced meningococcal vaccines is, therefore, very concerning. More generally, penicillin is prescribed in emergency situations when invasive meningococcal disease is suspected and ciprofloxacin is used as post-exposure prophylaxis to clear nasopharyngeal carriage.

### A call for vigilance

This is the first report of the US_NmUC strain outside of the US. Although the numbers are low, it is noteworthy that the strain has, thus far, only been observed in MSM in the UK rather than in heterosexual males, as was predominantly the case in the US. The increasing burden of gonorrhoea and emerging resistance to last-line antibiotics is already a major global threat and meningococci could follow the same trajectory. In the UK, presumptive gonorrhoea, as indicated by clinical features and Gram stain, is prescribed on-the-day anti-gonococcal treatment and partner notification is initiated [2]. Immediately-indistinguishable meningococcal infections presenting as such will also be covered by anti-gonococcal treatment, however, patients subsequently found to culture only meningococci (at least 24 hours later) would not usually be recalled, partner notification would not be done post-hoc, and a test of cure would not be done. Thus, if the UK and countries further afield are to avoid an outbreak similar to that seen in the US, more intensive management and follow up as per gonococcal disease may be indicated. Clinicians, microbiologists and public health teams, therefore need to remain vigilant and consider developing surveillance systems to include anogenital meningococci.
